# *Mycoplasma salivarium* as a Dominant Coloniser of Fanconi Anaemia Associated Oral Carcinoma

**DOI:** 10.1371/journal.pone.0092297

**Published:** 2014-03-18

**Authors:** Birgit Henrich, Madis Rumming, Alexander Sczyrba, Eunike Velleuer, Ralf Dietrich, Wolfgang Gerlach, Michael Gombert, Sebastian Rahn, Jens Stoye, Arndt Borkhardt, Ute Fischer

**Affiliations:** 1 Institute of Medical Microbiology and Hospital Hygiene, Medical Faculty, Heinrich Heine University, Düsseldorf, Germany; 2 Department of Paediatric Oncology, Hematology and Clinical Immunology, Center for Child and Adolescent Health, Medical Faculty, Heinrich Heine University, Düsseldorf, Germany; 3 Computational Metagenomics, Faculty of Technology, Center for Biotechnology, Bielefeld University, Bielefeld, Germany; 4 German Fanconi-Anemia-Help e.V., Unna, Germany; 5 Genome Informatics, Faculty of Technology, Center for Biotechnology, Bielefeld University, Bielefeld, Germany; University of Hawaii Cancer Center, United States of America

## Abstract

*Mycoplasma salivarium* belongs to the class of the smallest self-replicating *Tenericutes* and is predominantly found in the oral cavity of humans. In general it is considered as a non-pathogenic commensal. However, some reports point to an association with human diseases. *M. salivarium* was found e.g. as causative agent of a submasseteric abscess, in necrotic dental pulp, in brain abscess and clogged biliary stent. Here we describe the detection of *M. salivarium* on the surface of a squamous cell carcinoma of the tongue of a patient with Fanconi anaemia (FA). FA is an inherited bone marrow failure syndrome based on defective DNA-repair that increases the risk of carcinomas especially oral squamous cell carcinoma. Employing high coverage, massive parallel Roche/454-next-generation-sequencing of 16S rRNA gene amplicons we analysed the oral microbiome of this FA patient in comparison to that of an FA patient with a benign leukoplakia and five healthy individuals. The microbiota of the FA patient with leukoplakia correlated well with that of the healthy controls. A dominance of *Streptococcus*, *Veillonella* and *Neisseria* species was typically observed. In contrast, the microbiome of the cancer bearing FA patient was dominated by *Pseudomonas aeruginosa* at the healthy sites, which changed to a predominance of 98% *M. salivarium* on the tumour surface. Quantification of the mycoplasma load in five healthy, two tumour- and two leukoplakia-FA patients by TaqMan-PCR confirmed the prevalence of *M. salivarium* at the tumour sites. These new findings suggest that this mycoplasma species with its reduced coding capacity found ideal breeding grounds at the tumour sites. Interestingly, the oral cavity of all FA patients and especially samples at the tumour sites were in addition positive for *Candida albicans*. It remains to be elucidated in further studies whether *M. salivarium* can be used as a predictive biomarker for tumour development in these patients.

## Introduction

Human microbiomes represent complex, site-specific spectra of bacteria, fungi, and archaea, whose compositions are determined but also dependent on the state of health of the colonised individual. The microbiome of the gut is essential for food metabolism and uptake, whereas the oral microbiome preserves the physical integrity within the oral cavity and is functionally different from the gut environment [1]. Only around 50% of oral microorganisms can be cultivated and studied employing classical biochemical techniques at present. Next generation sequencing (NGS) of variable regions in the gene encoding the 16S rRNA first enabled in-depth, cultivation independent studies of the oral microbiomes [2]–[6]. Nine variable regions in the 16S rDNA can be used which differ in their potential to discriminate bacterial species [7], [8]. For instance, to answer the question how the oral microbiome of the saliva is composed in healthy people, Roche/454-next-generation-sequencing of amplicon libraries comprising the V1-V2 variable region of the 16S rDNA was employed that had been shown to be appropriate to achieve taxonomic assignment for a wide range of bacterial genera investigated [7], [8]. Besides unravelling the general composition of the microbial community, Costello and coworkers showed in 2009 that an individual's oral microbiome is stable over time by comparing samples taken on four different occasions. The group of Zaura compared the microbiomes from intra-oral sites of three systemically and orally healthy individuals employing the V5-V6 region of the 16S rDNA [9]. They hypothesized a core oral microbiome to be present in health with the predominant taxa/phyla belonging to Firmicutes, Proteobacteria, Actinobacteria, Bacteriodetes and Fusobacteria based on their findings of a great proportion of similar amplicon reads found in all subjects. Depending on the site of colonisation, the composition of oral microbiomes differs. Diaz and coworkers analysed bacterial communities in saliva and buccal mucosa and found that inter-subject variability was lower than differences between saliva and mucosal communities with high abundance of *Streptococcus mitis* and *Gemella haemolysans* predominantly found in the mucosa [10].

Variability of the oral microflora has been demonstrated to relate to oral diseases, too. In endodontic infections, Li and coworkers found *Bacteroidetes* as the most prevalent bacterial phylum in infected root canal spaces [11]. The group of Hsiao characterized the site-dependent microbiomes in endodontic infections and published that *Prevotella*, which belongs to the Bacteroidetes, and Fusobacteria were most abundant in the oral cavity whereas the Firmicutes: *Granulicatella adiacens*, *Eubacterium yurii*, and *Streptococcus mitis*; and the Bacteroidetes: *Prevotella melaninogenica* and *Prevotella salivae*; were over-represented in diseased tissues of root and abscesses [12]. *Porphyromonas gingivalis*, *Tannerella forsythia*, and *Actinobacillus actinomycetemcomitans* were characterised as contributing pathogens in periodontitis [13].

Besides infectious diseases, variability of the oral microflora also related to oral cancers. In the saliva of patients with oral squamous cell carcinoma (OSCC), high levels of facultative oral streptococci were observed [14] and members of eight phyla of bacteria were detected by using V4-V5 16S rDNA based 454 parallel DNA sequencing [15], [16]. The majority of identified amplicon reads corresponded to Firmicutes and Bacteroidetes and 67% of the reads to various as yet uncultured or unclassified bacteria. Interestingly, a low amount of reads in the saliva of OSCC patients belonged to mycoplasma (Tenericutes) (<0.5%), but none were detected in the saliva of the control group.

Mager and coworkers proposed in 2005 that the salivary microbiota can function as a diagnostic indicator of oral cancer. In a comparative analysis of the saliva of healthy people and patients suffering from OSCC they found that *Prevotella melaninogenica* and *Capnocytophaga gingivalis* of the Bacteroidetes and *Streptococcus mitis* of the Firmicutes were highly increased in the saliva of OSCC patients in contrast to healthy controls [17].

Fanconi anaemia (FA), which is a rare chromosomal instability disorder due to germ-line-mutations in specific genes involved in DNA repair, is clinically characterized by congenital malformations, progressive bone marrow failure and an increased risk of malignancies especially squamous cell carcinomas of the head and neck (HNSCC) at a relatively young age [18]. In addition to the clinical manifestations, chromosome breakage analyses are routinely employed for the diagnosis of the disease. Because of drastic side-effects due to the defective DNA repair, chemotherapy is generally not suitable and radiation goes along with a lot of side effects. Surgery is the most effective treatment of OSCC in FA patients, but requires an early detection of the malignant lesions [19]. In the present study, the oral microbiome was evaluated as an early indicator of cancerogenesis in such patients. To this end, microbiomes at distinct sites of the oral cavity from patients with Fanconi anaemia and healthy probands were comparatively analysed using next generation sequencing of the 16S rDNA V1-V2 region and real time PCR for quantifying prominent microorganisms such as *M. salivarium*.

## Results

**Figure 1:**
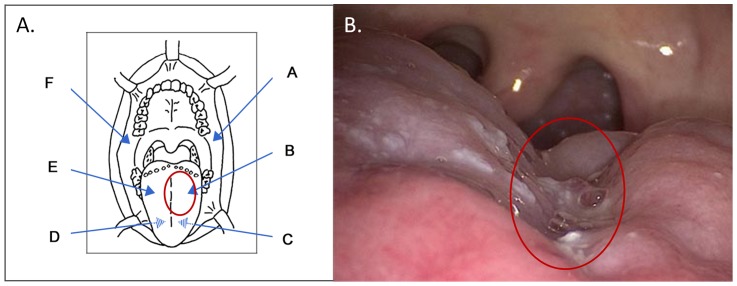
Oral cavity and sites of sampling. A. Anatomic chart of the oral cavity with arrows indicating the sites of sampling (A to F) and a red circle labelling the tumour region of patient FA_T1. Samples C and D were collected from the ventral portion of the tongue (indicated by arrows with hatched centres). B. Photography of the tumour on the FA_T1 patient's left side of the tongue encroaching the centre line. The red circle indicates the sampling site of the tumour region as described in Figure 1, A.

Following the hypothesis that the development of oral squamous cell carcinoma is accompanied by a preceding change of the microbiome-composition at the respective cell surface, oral swabs were collected from FA patients according to the anatomic chart as shown in Figure 1, A.

In a retrospective analysis, four samples of a 41 year old FA-patient (sample set FA_T1) were analysed, which corresponded to the surfaces of an oral squamous cell carcinoma of the tongue (swab B; see Figure 1, A and B), of the neighbouring gingival tissue (swab A) and two from both corresponding contralateral sites (swabs E and F). The samples of the tumour patient FA_T1 were compared to samples of another FA-patient who harboured a benign oral lesion (leukoplakia, sample set FA_L1) and samples of a control group of five healthy individuals (samples H1-H5). The sample sites of FA_L1 were the same as of FA_T1, the samples from the control group were all taken from site B. After preparation of genomic DNA, a high coverage, massive parallel Roche/454-next-generation-sequencing approach of the V1-V2 variable 16S rDNA region was used to obtain a total of 808,308 raw reads for 30 different samples with unique barcodes. After quality filtering in the de-multiplexing step, 272,152 sequence reads remained. From those 30 samples, thirteen samples (H1-H5, site B, FA_L1, sites A, B, E, F and FA_T1, sites A, B, E, F) belonged to this study. Table 1 gives an overview of the total number of sequence reads per sample. Using the NGS analysis software pipeline QIIME [20] 4,569 Operational Taxonomic Unit (OUT) clusters were built from all 272,152 sequences. The OTUs were subjected to further chimeric OTU filtering and taxonomical classification. 1,010 OTUs that were identified to be chimeric were excluded, which resulted only in a small change in fractions on all taxonomical levels between 0 - 0.5%. 15 taxonomic families were identified. Each accounted for at least 4% of the microbiota in one of the thirteen samples.

**Table 1:** Total number of reads generated per sample.

Sample set	Sample Site	Total Number of Reads	Barcode
**H1**	B	10,595	TACTGAGCTA
**H2**	B	8,230	CGAGAGATAC
**H3**	B	7,742	TCACGTACTA
**H4**	B	18,730	AGCACTGTAG
**H5**	B	10,590	TCTACGTAGC
**FA_L1**	A	5,342	ACATACGCGT
**FA_L1**	B	7,719	TCGTCGCTCG
**FA_L1**	E	12,042	TAGAGACGAG
**FA_L1**	F	10,923	TACTCTCGTG
**FA_T1**	A	8,677	AGACTATACT
**FA_T1**	B	7,229	ACTGTACAGT
**FA_T1**	E	5,741	ACTACTATGT
**FA_T1**	F	3,929	ACGCGAGTAT

**Figure 2:**
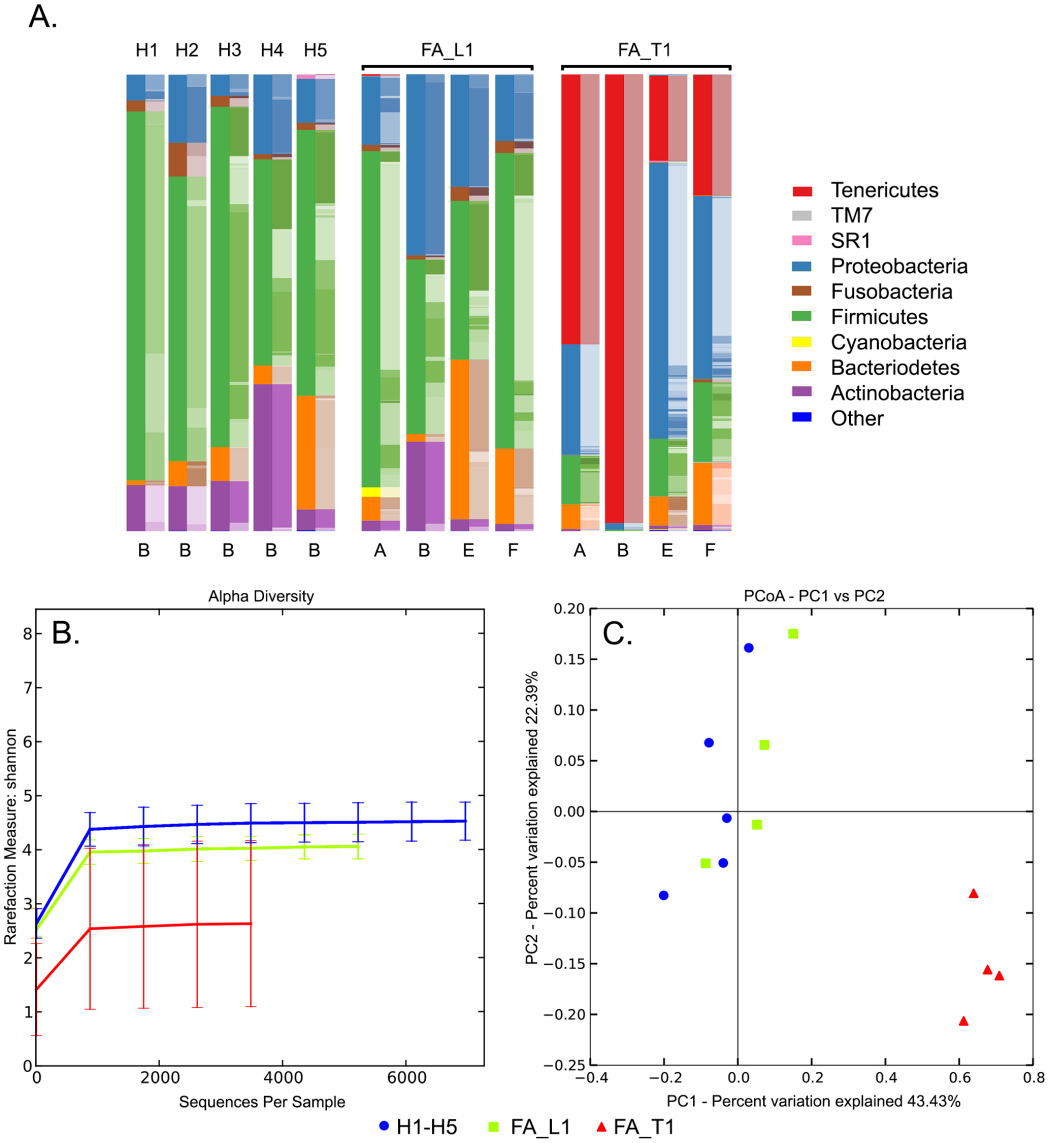
Microbiota detected by NGS on the surface of distinct oral sites of the analysed cohort. DNA of samples, which derived from site B of five healthy probands (H1-H5) and sites A, B, E and F of two FA patients (FA_L1 with a leukoplakia at site F and FA_T1 with a tumour at site B), was subjected to V1-V2 16S rDNA based NGS analysis. A., phyla and families detected in the 13 samples. Families are indicated in shades of the same colour as the respective phylum; B., alpha diversity of all samples (H1-H5 in blue, FA_L1 in green and FA_T1 in red); C., beta diversity plot of all samples (H1-H5 as blue circles, FA_L1 as green squares and FA_T1 as red triangles).

Bacteroidetes, Firmicutes, Proteobacteria and Tenericutes represented the most common phyla in the microbiomes of the four samples of FA patient FA_T1 (Figure 2, A), with Tenericutes as the predominant phylum found on the tumour surface (sample B). In contrast, the phyla of Actinobacteria, Bacteriodetes, Firmicutes and Proteobacteria were common in the respective samples of FA patient FA_L1 and all healthy probands (at site B), with a dominance of Firmicutes and lack of Tenericutes.

As depicted in Figure 2, B the mean species diversity at a given sample site (alpha diversity) is very low in the microbiota of FA_T1 compared to the FA_L1 microbiota and the control group. The diversity index (Shannon index) that approaches zero at lowest diversity was calculated for the control group (4.17±0.34), FA_L1 (3.75±0.2) and FA_T1 (2.43±1.42). The alpha diversity indicates that the microbiota is getting less diverse the more the probands are moving into a tumourous state. This observation is also supported by a calculation of the mean species diversity between different habitats (sample sites), the beta diversity (Figure 2, C). Samples derived from FA_T1 form a low diversity cluster on their own, completely separated from H1-H5 and FA_L1. The distribution of the data points of H1-H5 compared to FA_L1 suggests that the beta diversity is supporting our hypothesis of less diversity in an environment that is moving into a tumourous state. Taking these observations of reduced diversity into account, the dominant species, *Mycoplasma salivarium* can be proposed as a cofactor of the tumourous state of proband FA_T1. The significance and correlation of this finding was confirmed by the mantle test directly on the taxonomic distance matrix and the tumour weighted sample/site-specific distance matrix (with an r-value of 0.91 and a p-value of 0.001, where r stands for correlation and p for significance).

**Figure 3:**
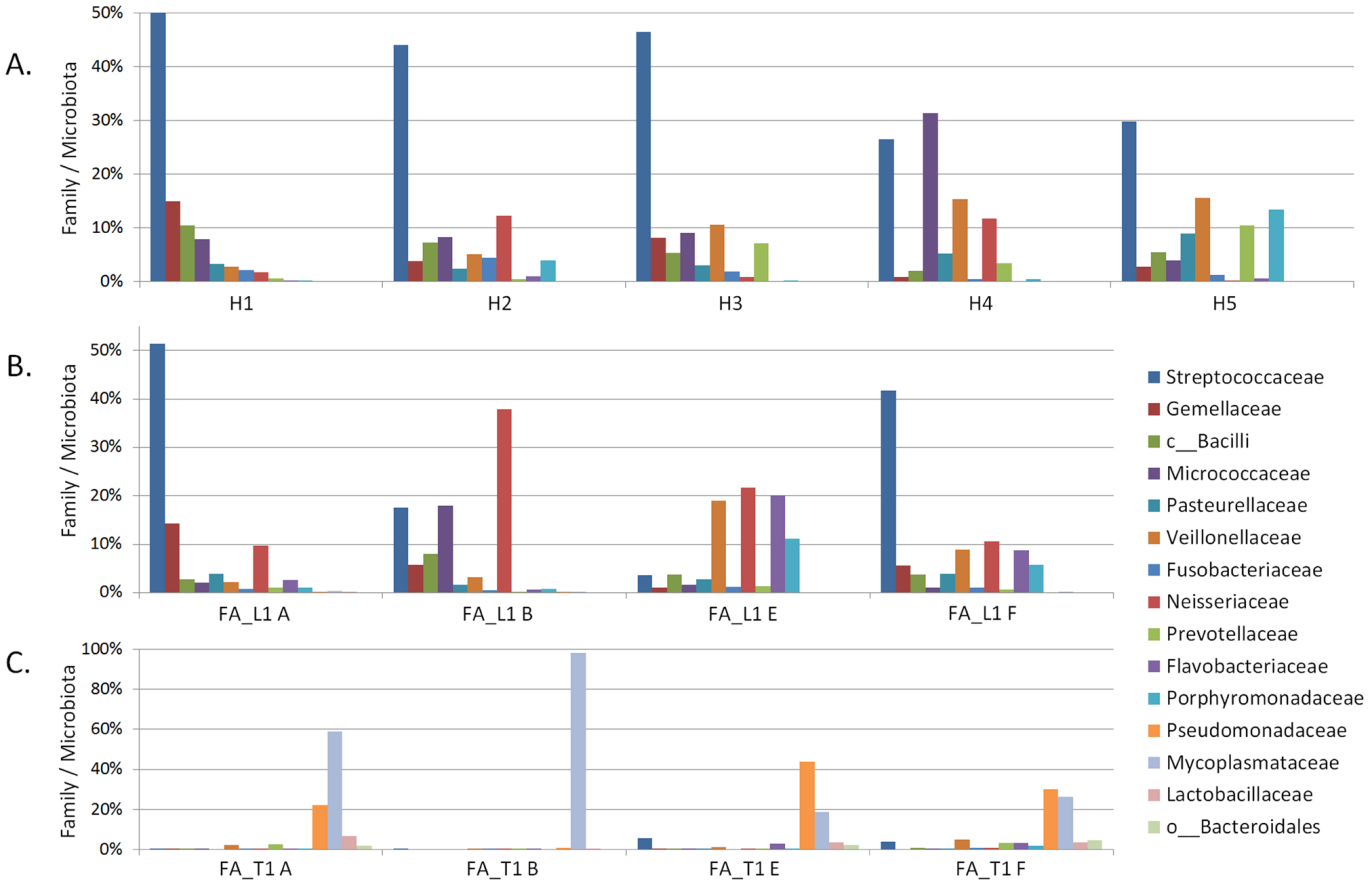
Dominant families detected by NGS after OTU clustering in the microbiotas of patients and healthy individuals. Frequency of families (in %) present in at least one of the 13 analysed samples analysed at a frequency >4%; A., site B of healthy probands H1-H5; B., sites A, B, E and F of FA patient FA_L1; and C., sites A, B, E and F of FA patient FA_T1.

Having a closer look at the respective taxonomic orders, reads classified as Mycoplasmatales comprised 98.2% of the microbiota on the tumour surface of patient FA_T1 and 59.1% of the gingival microbiota nearby the tumour. The variety of taxonomic orders found at the healthy site of tongue and gingiva of FA_T1 was much larger with the highest values for Pseudomonadales and Mycoplasmatales at tongue (43.8% and 18.7%, respectively) and gingiva (30.3% and 26.4%).The dominant families in the microbiota of healthy probands (Figure 3, A) and the FA patient FA_L1 (Figure 3, B) corresponded to one another. We found a predominance of *Streptococcaceae*, which belong to the Firmicutes, in nearly all of these samples and of *Neisseriaceae* and *Pasteurellaceae*, which belong to the Proteobacteria. In contrast, the percentage of *Streptococcaceae* was dramatically reduced in the microbiota of the tumour patient FA_T1 (Figure 3, C). *Mycoplasmataceae* dominated on the tumour surface (site B) and the adjacent gingiva (site A) and *Pseudomonadaceae* were frequent at all sites, except the tumour site.

Respective sequence reads of the predominant families were then clustered using the *SeqMan* program of the *LaserGene* software package. Highest homologous species were identified using the consensus sequences of each cluster (contig) in *MegaBLAST* analysis against the nucleotide collection database (nr/nt) of *BLASTN* (http://blast.ncbi.nlm.nih.gov/Blast.cgi [21]). Thus, it became obvious that in healthy probands and FA patient FA_L1 *Rothia mucilaginosa* was the dominant species of the Actinobacteria phylum, which was nearly absent in the tumour patient FA_T1 (see supplementary file of Table S1). *Prevotella melaninogenica* and *P. nanceiensis* of the Bacteroidetes phylum were also mainly found in the microbiota of healthy people and the leukoplakia patient whereas *P. salivae* and *Prevotella spp*. of different oral taxa dominated the gingival microbiota of the tumour patient. Of the Firmicutes, *Veillonella parvula* was comparably found in the samples of healthy and diseased probands, whereas *Selenomonas* spp. and *Megasphaera micronuformis* were only increased in the gingival samples of tumour patient FA_T1. Viridans-streptococci with members of the mitis-group such as *S. mitis*, *S. oralis* and *S. infantis* and *S. salivarius* of the salivarius-group comprise the main part of streptococci in healthy individuals and the leukoplakia patient. Higher levels of mutans-streptococci, such as *S. mutans* and *S. criceti*, and of *S. anginosus* were found only in samples of the tumour patient. A specific feature of the microbiota of the leukoplakia patient was found in the family of *Flavobacteriaceae* with *Capnocytophaga* species being increased at all sites. Highest levels of *C. sputigena* and *C. canimorsus* were found at the tongue next to the side of leukoplakia.

With the exception of a single *M. faucium* sequence read detected in one of the healthy individuals, *Mycoplasma salivarium* was the unique species of the *Mycoplasmataceae*, and *Pseudomonas aeruginosa* the main species of the *Pseudomonadaceae* detected at highest levels in the microbiota of the tumour patient. Thus, it is likely that within the proteobacterial phylum, the *Neisseriaceae*, with *N. flavescens* increased in healthy and leukoplakia individuals, have been displaced by *P. aeruginosa* in the tumour patient.

**Figure 4:**
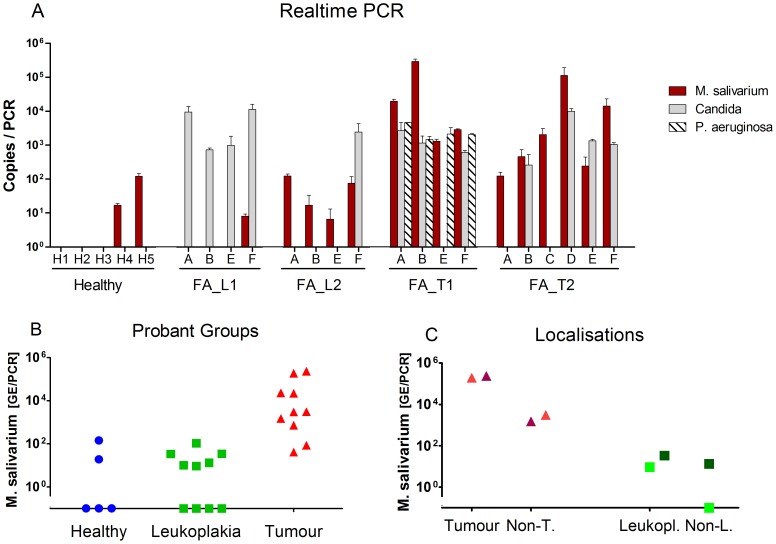
Real time PCRs for the detection of *M. salivarium*, *P. aeruginosa*, and *Candida*. DNA samples, which were derived from healthy probands (H1-H5, site B), FA-patients with leukoplakia (FA_L1 with a leukoplakia at site F and FA_ L2 with leukoplakia at site B) and FA patients with tumour (FA_T1 with a tumour at site B and FA_T2 with tumour at site D) were subjected to real time PCR in duplicates. A. TaqMan qPCR-derived copy numbers of *M. salivarium* (red), *P. aeruginosa* (hatched) and *Candida* (grey). Due to targeting a single copy gene, copy numbers correspond to genome equivalents for *M. salivarium* and *P. aeruginosa*. B., scatter plot of *M. salivarium* load in the different proband groups: Healthy (H1-H5, blue circles), samples of FA-patients with leukoplakia (FA_L1 and FA_L2, green squares) and with tumour (FA_T1 and FA_T2, red triangles). C., scatter plot of *M. salivarium* load of FA patients FA_T1 (dark red) and FA_T2 (red) at the tumour surface (Tumour) in relation to the contralateral non-tumour surface (Non-T.) and of the FA patients FA_L1 (green) and FA_L2 (dark green) at the site of leukoplakia (Leukopl.) in relation to the contralateral non-leukoplakia site (Non-L.).

TaqMan PCRs were used to quantify the bacterial load of *Pseudomonas aeruginosa* and *Mycoplasma salivarium* targeting the single copy genes *gyr*B and *rpo*B, respectively. As depicted in Figure 4, A in red, the results of the 16S rDNA survey were confirmed by *M. salivarium* qPCR, with the highest load of *M. salivarium* on the tumour surface of patient FA_T1 and lower amounts at the corresponding healthy sites of tongue and gingiva. For the TaqMan PCR analyses we included samples of a second FA tumour patient (FA_T2, sample set A,B,C,D,E and F with site D corresponding to the tumour surface), and a second leukoplakia patient (FA_L2, sample set A, B, E, F with leukoplakia at site B). For these two patients no NGS data were available. Interestingly, colonisation of both leukoplakia patients, FA_L1 and FA_L2, with *M. salivarium* was rare or of low concentration as in healthy probands (see Figure 4, B), whereas all samples of both tumour patients were *M. salivarium* positive with the highest load at the tumour surfaces. The *M.salivarium* load at the local sites of cellular alterations (tumour or leukoplakia) was compared with the respective contralateral sites of each FA-patient (Figure 4, C). The greatest differences in *M. salivarium* load were found in the tumour FA patients. Quantification of *P. aeruginosa*, which is shown in Figure 4, A, hatched, revealed that FA patient FA_T1 was the only one with a high load of this pathogen. All other FA patients and healthy probands were tested *P. aeruginosa*-negative.

As it had been known that the patient FA_T1 had have contracted oral mucositis for a long time and that oral candidiasis often impedes tumour detection at an early stage, DNA of all 23 samples were subjected to a *Candida*-specific qPCR, followed by melting curve analysis for product detection. As shown in Figure 4, A in grey, *Candida*-PCR was positive in most samples of FA patients FA_T1 and FA_T2, including the respective tumour regions B and D, and all samples of the FA patient FA_L1 with highest *Candida* load at the site F of leukoplakia. In contrast, samples of the healthy probands and samples A, B and E of FA patient L2 with leukoplakia at site B were *Candida*-negative. Products of *Candida*-PCR and of a confirmatory ITS1-ITS4- fungal PCR were sequenced by priming with ITS4 using the method of Sanger [22] and homologous species identified in BLAST analysis. All samples were positive for *Candida albicans* (90-99% homology).

## Discussion

Fanconi anaemia is associated with an increased risk to develop OSCC which is difficult to diagnose at an early stage of development by the use of minimal invasive procedures. Currently, short-period screening of the patients is one of the preventative measures. With the findings of Mager *et al.*, published in 2005 that the salivary microbiomes of OSCC patients differ from those of healthy people [17], a concept was formed that development of OSCC is accompanied by change of the microbiome-composition especially at the respective cell surface. To test this hypothesis, oral microbiomes were characterised by next generation sequencing and real time PCR. Four samples were of primary interest because they were collected from the surface of an oral squamous cell carcinoma of the root of the tongue, the adjacent gingiva and the contralateral (healthy) sites. These specimens were derived from a male patient born in 1970 who was diagnosed with Fanconi anaemia at the age of nine years. The advanced squamous cell carcinoma at the base of the tongue (cT3, cN2b, M0, G3) was diagnosed in July 2011. The centre line encroaching tumour (Figure 1, B) was inoperable with recidivating bleedings in pronounced thrombocytopenia. After tracheostomy, pneumonia-derived sepsis in August 2011 was treated with tacobactam/piperacillin. Palliative radiotherapy of the tumour was aborted in October 2011 due to severe complications of haemorrhage and mycositis. After suffering two pneumoniae-derived septicaemias in November with the detection of *Pseudomonas aeruginosa* and *Klebsiella pneumoniae* in tracheostomal smear and tracheal secretion, G-CSF therapy started. Antibiogram revealed selection of a highly resistant strain of *P. aeruginosa* in the second sepsis, probably due to piperacillin/tazobactam therapy. The patient died at the end of December 2011. As stated by a family member the patient had suffered from mucositis for longer times without being medicated other than using an oral Moronal (Nystatin) suspension.

The composition of the oral microbiome in each of the four samples of this FA patient FA_T1 differed from that of the proposed oral core microbiome of healthy people, which was defined by Zaura *et al*. to be mainly composed of Firmicutes, Proteobacteria, Actinobacteria, Bacteriodetes and Fusobacteria [9]. Interestingly, the microbiota of the FA patient with leukoplakia, FA_L1, corresponded to that of a healthy person, as those of the healthy probands included in the present investigation, too. In contrast, the oral microbiome of the FA patient with tumour (FA_T1) was dominated by *Tenericutes* and *Proteobacteria*, contained *Bacteroidetes* and *Firmicutes* but lacked higher amounts of *Actino-* and *Fusobacteria*. The dominance of the opportunistic pathogenic proteobacterium *Pseudomonas aeruginosa* in the oral cavity of the patient was not surprising, as the respiratory tract of patients with tracheostomy often becomes infected with *P. aeruginosa* [23], [24] and two episodes of *P. aeruginosa*-derived pneumonia and septicaemia were known. A study of He and coworkers demonstrated that the salivary microbiota of healthy humans is able to prevent the integration of pathogenic bacteria such as *P. aeruginosa* [25] suggesting that the oral microbiota of the immunocompromised FA patient must have already been misbalanced before to enable *P. aeruginosa* colonisation. Unfortunately, the antibiotic regime has led to the selection of a highly resistant *P. aeruginosa* strain difficult to eradicate.

The detection of high loads of *M. salivarium* in each sample of the tumour patients FA_T1 and FA_T2 with prevalence on the surface of the oral cancers was an unexpected finding, especially as *M. salivarium* was generally considered as a non-pathogenic inhabitant of the oral cavity [26]. Only in a few cases it has been considered to participate in oral and periodontal infections [27]–[30] or to be the causative agent of a submasseteric abscess [31]. Nevertheless, as *M. salivarium* is difficult to culture, it has thus been rarely looked for. Incidental findings identified *M. salivarium* as causative agent of disseminated infections, such as a chronic joint infection in a patient with hypogammaglobulinaemia [32]. *M. salivarium* was found in brain abscesses of two patients [33], in an occluded biliary stent in polymicrobial community with *Candida glabrata* [34] and as causative agent of a pleural empyema [35].

Characterisation of complex microbiomes by using molecular biological approaches such as NGS nowadays offers the opportunity to detect species that are uncultivable or difficult to culture such as *M. salivarium*. Having a closer look at the yet published 16S rDNA surveys only a few descriptions can be found about mycoplasmas taking part in oral microbiomes. Dewhirst and coworkers published in 2010 a comprehensive human oral microbiome database (HOMID) based on yet published 16S rDNA sequences. In their clone libraries they only found relative few mycoplasmas with representatives of *Mycoplasma hominis*, *Mycoplasma salivarium*, *Mycoplasma faucium* [36] and representatives of *Tenericutes* [G-1] sp. oral taxon 504, which were very deeply branching within the *Tenericutes*. In using the Human Oral Microbe Identification Microarray (HOMIM) *M. salivarium* was found in subgingival plaque samples of six of 47 subjects with refractory periodontitis [30]. *Mycoplasma* reads were found in the saliva of OSCC patients representing less than 0.5% of the microbiomes [15]. These findings do not suggest that *M. salivarium* is a predictive bio-marker of tumour development. However, one should keep in mind that analysis of the salivary microbiome is not specific for the tumour site and that *M. salivarium* is not a general coloniser of the oral cavity. Interestingly, all of the analysed FA patients were carriers of *M. salivarium*, but only two of five healthy individuals. Hooper and coworkers analysed OSCC samples by culturing and 16S-based PCR after elimination of the surface attached microbes. Within the tumour tissue they found that the majority of species were saccharolytic and aciduric, reflecting perhaps the selective nature of the acidic and hypoxic microenvironment found within tumours [37], [38]. However, in their study the presence of *M. salivarium* within the tissue was undetectable due to culture media used, which were not suitable for mycoplasma growth, and due to a serious mismatch of the Reverse primer E94 at its 3′-end (5′-GAAGGAGGTGWTCCARCC**g**CA-3′) hampering amplification of the 16S rDNA-region 5′-**A**AAGGAGGTGATCCATCC**C**CA-3′) of *M. salivarium* (Acc.-No: AY786574; nt 141-121).

Depending on the amplification conditions used in NGS, the ratio of each phylum differs. Lazarevic *et al.* demonstrated in 2012 that the amount of Tenericutes (*Mycoplasma*) virtually increases in the salivary microbiome when stepping the cycle number in 16S V1-V3-PCR from 20 up to 30 [39]. Thus, quantification of bacteria using NGS analyses seems to be hindered in more than one aspect. On the one hand the amplification conditions may shift the real ratio of microorganisms in a defined environment to something unrelated in the 16S rDNA amplicon based microbiota, and on the other hand, the amount of 16S rDNA derived amplicons is biased by the number of ribosomal operon structures of a bacterium. Whereas the genome of *Mycoplasma orale* harbours only one ribosomal operon structure, the genome of *Lactobacillus delbrueckii* harbours nine 16S rDNA copies [40], [41], which leads to an overestimation of *L. delbrueckii*. Thus, it seems to be difficult to quantify a species by a metagenome analysis. In the study presented here, the amounts of *M. salivarium* and *P. aeruginosa* were rechecked by TaqMan PCRs, each of those targeting a single copy gene. This approach confirmed a predominance of *M. salivarium* at the tumour sites. *P. aeruginosa*, exclusively found in the oral cavity of FA patient FA_T1, was not restricted to the tumour site, but detected at all analysed sites in the mouth with similar abundance.

The findings of this study that the oral microbiomes of the tumour FA patients were colonised by high levels of *M. salivarium* and *C. albicans* but, as shown in NGS analysis of FA patient FA_T1, only low levels of streptococci, are different to those of Tateda and Sasaki, who found high level of colonisation on OSCC with facultative oral streptococci relative to uninvolved mucosa [42], [43]. *S. mitis*, which is known to play a role in endodontic infections [8] and to be highly increased in the saliva of OSCC patients [17], was dramatically reduced in the gingival samples of the FA patient, FA_T1. Our findings in healthy individuals are in agreement with the data of Diaz and coworkers [10], who found *S. mitis* and *Gemella haemolysans* to be abundant on healthy mucosal surfaces [10] and suggested that depending on the degree of leukoplakia towards dysplasia and finally tumour, the oral microbiome may correspond to that of a healthy person, like the leukoplakia FA patient FA_L1, or already indicate misbalance in microbial diversity like in tumour FA-Patient FA_T1.

Panghal *et al.* described in 2012 incidence and risk factors for infection in oral cancer patients undergoing different therapies [44]. They found that the predominant gram-negative *P. aeruginosa* and *Klebsiella pneumoniae* were isolated from blood of cases treated by radiotherapy and from the oral cavity of cases treated by chemotherapy, respectively. *C. albicans* was found as most predominant fungi of the oral cavity after radio- and chemotherapy. Thus, palliative radiotherapy of the tongue tumour - although preliminarily aborted due to severe complications – might have been an additional co-factor for growth of *P. aeruginosa* and *C. albicans* in the oral cavity of the FA patient. Interactions of *C. albicans*, a fungal coloniser of the oral cavity, with *P. aeruginosa* and *S. aureus* are well known [45] and colonisation of the respiratory tract with *C. albicans* seems to promote *P.aeruginosa* associated ventilator-associated pneumonia [46]. The information that *M. salivarium* was detected in a microbial community with *Candida glabrata* in the biofilm of an occluded biliary stent [34] may lead to the hypothesis that even *C. albicans* may promote colonisation with *M. salivarium* or vice versa. The new findings that *C. albicans* was detected in each of the four FA patients tested and in both tumour patients in bacterial community with high loads of *M. salivarium* at the tumour surface may point to a pathophysiological role of both microorganisms in FA patients.

At the moment it is not clear whether colonisation with *M. salivarium* is a prerequisite of tumour development or rather the result of altered tissue morphology that clears the way for *M. salivarium* growth. In both cases *M. salivarium* may be an interesting candidate diagnostic biomarker. In further long-term studies the potential of *M. salivarium* as a candidate OSCC biomarker will be evaluated. In comprehensive investigations, oral microbiomes will be analysed at different sites in the mouth (see Figure 1, A) in higher numbers of still healthy FA patients to define their individual microbiome pattern before disease. Periodic screening will then enable the identification of alterations in the polymicrobial composition and, in case of cancer development, the definition of biomarkers for tumour development. In addition, it will be interesting to analyse oral microbiomes of OSCC patients without Fanconi anaemia to clarify whether these biomarkers are restricted to oral cancer of FA patients or to oral cancer in general. Next generation sequencing is a useful methodological approach to identify species such as *M. salivarium*, which would never have been looked for, and should also be adopted for the detection of fungi such as *Candida albicans*.

## Materials and Methods

### Ethics statement

Swab samples of the patients were collected during the course of normal treatment. All subjects provided written informed consent to participate in this study. The consent procedure was part of the protocol sent to the local ethics committee of the Medical Faculty of the Heinrich-Heine University Düsseldorf who approved the study for analysing the significance of the local microbiome on the pathogenesis of oral FA carcinoma (Study-No.: 3613).

### Subjects

Five healthy individuals were included in the present investigation; three females (H1, H2 and H5, at the age of 42, 32 and 43, respectively) and two males (H3, and H4, at the age of 37 and 33). FA Patient FA_T1, whose anamnesis is extensively described in the discussion section, did not undergo allogeneic hematopoietic stem cell transplantation (HSCT) whereas all other FA patients were transplanted: the 27 year-old FA patients, female FA_T2 and male FA_L1 at the age of five, and the 33 year old female FA patient FA_L2 at the age of 10. None of the patients FA-T2, FA_L1 and FA_L2 did receive chemo- or radiotherapy for any cancer at any time prior to swab collection.

### Sampling and total DNA extraction

Swabs were collected from distinct sites of the mouth (see Figure 1) and stored in 500 μl of CytoLyt solution (Cytyc Corporation, Massachusetts, USA). Total genomic DNA was isolated using the DNA Blood Kit (Qiagen, Hilden, Germany). All intact cells (bacterial, fungal and human) were collected by centrifugation (10 min at 5,000×g), resuspended in enzyme solution (20 mg/ml lysozyme; 20 mM Tris/HCl, pH 8.0; 2 mM EDTA; 1.2% Triton) and incubated for at least 30 min at 37°C. 20 μl Proteinase K (>600 mU/ml) and 200 μl Buffer AL (QIAmp Blood Kit, QIAGEN) were added and incubated at 56°C for 30 min and for 15 min at 95°C. DNA isolation then followed the standard protocol for DNA preparation from tissues according to the recommendations of the manufacturer. DNA preparations were stored at −20°C until use.

### Real time PCR

Real time PCR assays for the detection of *Candida* DNA were carried out according to the method of Schabereiter-Gurtner *et al.* [47] in using Can-F (5′-CCT GTT TGA GCG TCR TTT-3‘) and ITS-4 (5‘-TCC TCC GCT TAT TGA TAT GC-3‘) [48] for *Candida* amplification. Real time-PCR was carried out in a total volume of 30 μl consisting of 1x MesaGreen qPCR MasterMix Plus for SYBR Assay (containing Buffer, dNTPs (including dUTP), Meteor Taq DNA Polymerase, 4 mM MgCl_2_, SYBR Green I, stabilizers and passive reference (RT-SY2X-06+WOU); Eurogentec, Seraing, Belgium), 300 nM each forward and reverse primer and 2 μl of template DNA. Concurrent amplification of 10^5^ and 10^2^ copies of pGemT-cloned ITS1-ITS4 amplicons enabled quantification of *Candida* load. Each positive *Candida* detection was verified in ITS1-ITS4-primed PCR [48] followed by sequencing [22] and BLAST analysis [21]. Cycling conditions of both PCRs were as follows: 2 min at 95°C followed by 40 cycles of 30 s at 95°C, 30 s at 55°C and 1 min at 72°C. Subsequent melting point analysis followed after 15 s at 95°C and 1 min at 60°C from 65°C to 95°C with an increment of 0.5°C for 15 s and plate read.

### TaqMan PCR

*In house* TaqMan-PCRs were carried out in a total volume of 25 μl consisting of 1× Eurogentec qPCR MasterMix without ROX (containing buffer, dNTPs (including dUTP), HotGoldStar DNA polymerase, 5 mM MgCl_2_,Uracil-N-glycosylase and stabilizers ((RT-QP2X-03NR, Eurogentec), 300 nM each forward and reverse primer, 200 nM labelled probe and 2.5 μl of template DNA. The *rpo*B-based *M. salivarium* TaqMan PCR was carried out with the primers Msal-F (5′-CCG TCA AAT GAT TTC GAT TGC-3′) and Msal-R (5′-GAA CTG CTT GAC GTT GCA TGT T-3′) and probe Msal-T (5′-HEX-ATG ATG CTA ACC GTG CGC TTA TGG GTG-BHQ1-3′) [34]. The gyrB-based *P. aeruginosa* TaqMan-PCR was carried out with the primer pair Paer-F (5′-CCT GAC CAT CCG TCG CCA CAA C-3′) and Paer-R (5′-CGC AGC AGG ATG CCG ACG CC-3′) and the probe Paer-T (5′-FAM-CCG TGG TGG TAG ACC TGT TCC CAG ACC-BHQ1-3′) generating a 222 bp amplicon. Amplicon carrying plasmids (pGemT-Msali and pGemT-Paeru) were used in three concentrations (10^5^, 10^3^ and 10^2^ copies /μl) as quantification standards. Each sample was analysed in duplicates. Cycling, fluorescent data collection and analysis were carried out with an iCycler from BioRad (BioRad Laboratories, Munich, Germany) according to the manufacturer's instructions.

### Next-Generation-Sequencing (NGS)

Primers used in this study (forward primer: 5‘-GCC TTG CCA GCC CGC TCA GTC AGA GTT TGA TCC TGG CTC AG-3‘ and reverse primer: 5‘-GCC TCC CTC GCG CCA TCA GNN NNN NNN NNN NCA TGC TGC CTC CCG TAG GAG T -3‘) targeted the conserved region of the 16S rDNA flanking the V1-V2 hypervariable regions [49], [50]. The forward primer contained part of the Roche/454 primer B sequence (GCC TTG CCA GCC CGC), a key sequence (TCAG), the bacterial primer 27F and a two base pair („TC“) linker sequence. The reverse primer contained part of the Roche/454 primer A sequence (GCC TCC CTC GCG CCA), a key sequence (TCAG), a barcode sequence (indicated by “NNNNNNNNNNNN”), the bacterial primer 338R and a “CA”-linker.

25 μl PCR reactions were carried out in triplicates per sample with 0.4 μM each primer, 1 μl template DNA and 1x Platinum PCR SuperMix (22 U/ml complexed recombinant Taq DNA polymerase with Platinum Taq Antibody, 22 mM Tris-HCl (pH 8.4), 55 mM KCl, 1.65 mM MgCl_2_, 220 μM dGTP, 220 μM dATP, 220 μM dTTP, 220 μM dCTP, and stabilizers; Invitrogen/Life Technologies, Darmstadt, Germany). Cycling conditions consisted of an initial denaturation step at 94°C for 3 min followed by 35 cycles of 94°C for 45 s, 50°C for 30 s and 72°C for 90 s, terminated by a final extension step at 72°C for 10 min.

Triplicates were pooled and purified using the Gel Extraction Kit (Qiagen). Quality and quantity was assessed using the Quant-iT PicoGreen dsDNA kit (Invitrogen). Purified amplicons were combined in equimolar ratios into a single tube. Pyrosequencing was carried out using primer A and B on a Roche/454 Life Sciences Genome Sequencer FLX instrument according to the recommendations of the manufacturer (Roche, Mannheim, Germany).

Raw sequence data were deposited at the European Nucleotide Archive (ENA) under the study accession PRJEB5069 (http://www.ebi.ac.uk./ena/data/view/PRJEB5069).

### Analysis of NGS data using QIIME

The analysis of the Roche/454 NGS data was performed with the *QIIME* NGS analysis pipeline, which is a general purpose collection of tools and scripts covering the needs of all necessary steps from raw data processing over data normalization, clustering, taxonomical classification to statistics and its visualization [20].

#### Demultiplexing and quality control

QIIME contains a ready-made workflow for 454 sequenced 16S rDNA beginning with cleaning and mapping the input data with respect to the barcode, linker primer and the reverse primer sequence (Figure S1). The demultiplexing step removed the primer sequences and assigned reads to samples identified by the correspondent barcode. Quality filtering was also performed with the following default parameters: quality phred score of 25, min/max read length of 200/1000, no ambiguous bases and mismatches in the primer sequences.

#### OTU creation and taxonomical classification

Clustering was performed with *uclust* [51], a seed based clustering approach, which was run with a minimal sequence identity of 97%. The output was a set of Operational Taxonomic Units (OTUs) with each OTU representing a single species [as specified with the 97% internal sequence identity].

Subsequently, representatives from every OTU cluster were aligned with *PyNAST* [52] for chimera depletion with *ChimeraSlayer* of the Microbiome Utilities Portal of the Broad Institute, (http://microbiomeutil.sourceforge.net/ [53]). After removing the chimeric OTU clusters, the remaining representatives were used for taxonomic classification with the *RDP classifier* [54] and the *Greengenes* OTU database (http://greengenes.secondgenome.com/; Version: 12_10, 97% identity level [species level] [55]) with the default confidence factor of 80%. Reads belonging to a taxonomical family with a minimal total proportion of 4% in one of the four samples were clustered in using the *SeqMan* program of the *LaserGene* software package (Version 6; 97% identity level; DNA Star, Madison, WI, USA). Highest homologous species were identified in using the resulting consensus sequences in *MegaBLAST* analysis against the nucleotide collection database (nr/nt) of *BLASTN* (http://blast.ncbi.nlm.nih.gov/Blast.cgi [21]) with an exclusion of uncultured /environmental sample sequences. The species of the *Streptococcaceae* were identified using *vmatch* [56] by comparing the input sequences with the *Silva 115 release 16S* reference set [57].

Genome equivalents were calculated from the number of V1-V2 sequence reads of *M. salivarium* and *P. aeruginosa* based on the copies of 16S rDNA per genome. The genome of *P. aeruginosa* is known to carry four 16S rDNA copies [40] and the *M. salivarium* genome carries one 16S rDNA gene (https://img.jgi.doe.gov/cgi-bin/m/main.cgi?taxon_oid=2534682365).

### Computation of alpha and beta diversity

The calculation of the alpha diversity was performed by using the QIIME alpha rarefaction workflow. The five H1-H5 samples from sample site B were pooled as *control group*, the four samples (sites A, B, E, F) from FA_L1 as *FA_L1 group* and the four samples (sites A, B, E, F) from FA_T1 as *FA_T1 group*. The pooled samples were then compared with the Shannon index [58].

Phylogenetic beta diversity analysis was accomplished via the weighted *UniFrac* metric [59] and the OTU based taxonomical representative tree. From every sample 1000 sequences were taken into computation of the beta diversity. The PCoA plot was generated from the principal coordinates 1 and 2. The correlation was tested using the mantle test comparing the weighted *UniFrac* matrix with a proband/sample site-specific tumour weight matrix. H1-H5 were weighted as 0, FA_L1 A, B, E as 10 and FA_L1 F as 15, FA_T1 A, E, F as 75 and FA_T1 B as 100. Principal coordinates are a generalisation of the underlying taxonomical information of sample sets with a minimal loss of information. From the input data principal coordinates are generated, which represent a transformation of the high dimensional taxonomical information into the lower dimensional (generalised) feature space of principal coordinates.

## Supporting Information

Figure S1 **Common amplicon structure.** The target sequence (V1-V2 of the 16S rDNA) is amplified using sequence specific forward (27F) and reverse (338R) primers. Amplicons belonging to a specific sample are identified by an integrated unique barcode sequence. The flanking adapter sequences A and B are sequencer –specific primer sequences. Linker sequences are introduced to provide greater flexibility. The resulting common amplicon structure is depicted.
(TIF)

Table S1 Species of the dominant families detected by NGS and their ratio in the microbiotas of FA patients and healthy individuals.
(XLS)
