# Supplementary figures and images for: Mycoplasma salivarium as a Dominant Coloniser of Fanconi Anaemia Associated Oral Carcinoma
Source: PLoS One. 2014 Mar 18;9(3):e92297. doi: 10.1371/journal.pone.0092297 (PMC3958540; doi:10.1371/journal.pone.0092297)

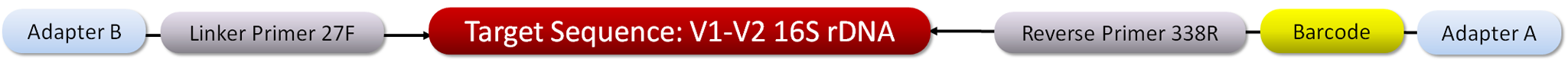

Supplement: Figure S1 — Common amplicon structure. The target sequence (V1-V2 of the 16S rDNA) is amplified using sequence specific forward (27F) and reverse (338R) primers. Amplicons belonging to a specific sample are identified by an integrated unique barcode sequence. The flanking adapter sequences A and B are sequencer –specific primer sequences. Linker sequences are introduced to provide greater flexibility. The resulting common amplicon structure is depicted. (TIF) [file pone.0092297.s001.tif]
